# Application Prospect of Artificial Intelligence in Rehabilitation and Management of Myasthenia Gravis

**DOI:** 10.1155/2021/5592472

**Published:** 2021-03-04

**Authors:** Ying Zhang, Hongmei Yu, Rui Dong, Xuan Ji, Fujun Li

**Affiliations:** ^1^Department of Neurology, The Second Affiliated Hospital of Harbin Medical University, Harbin, Heilongjiang, China; ^2^Department of General Surgery, The Second Affiliated Hospital of Harbin Medical University, Harbin, Heilongjiang, China

## Abstract

Myasthenia gravis (MG) is a chronic autoimmune disease of the nervous system, which is still incurable. In recent years, with the progress of immunosuppressive and supportive treatment, the therapeutic effect of MG in the acute stage is satisfactory, and the mortality rate has been greatly reduced. However, there is still no consensus on how to conduct long-term management of stable MG, such as guiding patients to identify relapses, practice exercise, return to work and school, etc. In the international consensus guidance for management of myasthenia gravis published by the Myasthenia Gravis Foundation of America (MGFA) in 2020, for the first time, “the role of physical training/exercise in MG” was identified as the topic of discussion. Finally, due to a lack of high-quality evidence on physical training/exercise in patients with MG, the topic was excluded after the literature review. Therefore, this paper reviewed the current status of MG rehabilitation research and the difficulties faced by stable MG patients in self-management. It is suggested that we should take advantage of artificial intelligence (AI) and leverage it to develop the data-driven decision support platforms for MG management which can be used for adverse event monitoring, disease education, chronic management, and a wide variety of data collection and analysis.

## 1. Introduction

Myasthenia gravis (MG) is a chronic autoimmune disease affecting the postsynaptic membrane at the neuromuscular junction thereby obstructing nerve impulse transmission [1, 2]. The prevalence rate (PR) of MG is 15 to 179 cases per million, estimated pooled PR is 77.7; and the incidence rate (IR) is 1.7 to 21.3 cases per million person-years, estimated pooled IR is 5.3 [3]. MG treatment traditionally includes the symptomatic treatment by acetylcholinesterase inhibitors, thymectomy, steroids and/or nonsteroidal immunosuppressive therapy, intravenous immunoglobulin (IVIg), or plasmapheresis. Advancements in therapeutics have significantly reduced MG-associated mortality [4, 5]. However, the disease still affects most patients' daily functional activities and reduces their quality of life [6–9]. Since it is difficult for patients with MG to achieve complete stable response (CSR), the current international consensus has proposed minimum performance state (MMS) or better as the treatment goal of MG [10]. Therefore, the chronic management of MG includes complex pharmacotherapy and lifestyle-related activities, which must be optimized to improve the quality of life in patients and enable as many patients as possible to achieve MMS or better treatment goals. In the past few years, more and more AI-based software and hardware have been developed for the management and clinical research of chronic diseases. In this review, we introduce the successful experience of AI in several chronic disease management and discuss the possible application of AI in the rehabilitation and management of MG in the future.

## 2. Current Status and Dilemma of Rehabilitation and Management in MG

For a long time, physicians have paid more attention to the treatment of acute symptoms in MG; however, the potentially prolonged course of the disease remains neglected. In many MG patients, even if symptoms are improved by standardized treatment after onset, they will soon be plagued again by disease recurrence or drug-related adverse events. Here are several common reasons: (1) relapse as a consequence of early withdrawal of medication, undertaken due to resolution of symptoms; (2) adverse events caused by failure to regularly monitor the safety of medication; (3) other chronic diseases caused by long-term use of glucocorticoids and immunosuppressive drugs; and (4) relapse of MG for unknown causes. With the prolongation and fluctuation of the disease, MG patients are often puzzled over the period of their treatment. “How much and how long should I take my medicine? What should I pay attention to in my daily life? Can I do physical exercise? When can I go to work? Why is my sleep getting worse?” Even in the recently published consensus guidance for management [11], the expert group did not give clear recommendations for the management of stable MG patients. Some studies have shown that most MG patients are often restricted when engaging in social activities, such as entertainment or work. Patients present with depression, anxiety, social reclusiveness, and frustration to a certain extent, arising from their MG [12–14]. Therefore, treatment strategies should also include the long-term management of MG and aim towards an improved quality of life and mental health [15].

The preventive benefits of physical exercise in various chronic diseases such as stroke, heart diseases, and cancer are well known, and its role in promoting positive psychological effects is beginning to be widely accepted at the same time [16–18]. Physical exercise has even been compared to drug therapy and has been therefore recommended as part of the management of many chronic diseases.

Traditionally, it was believed that physical training/exercise exacerbated symptoms for MG patients. However, the current general opinion supports that MG patients can benefit from physical exercise, which the evidences coming from a small number of physical exercise clinical trials. In a stratified study by Rahbek et al. [19], patients were assigned randomly to either the aerobic training (AT) group or the progressive resistance training (RT) group intervention over 8 weeks. Primary results showed that MG patients were well tolerated to both types of physical intervention within a specified time period (i.e., 8 weeks). Secondary results showed that muscle strength and functional capacity improved in the RT group, compared with no change in the AT group. A prospective study, by Westerberg et al. [20], supervised AT and RT twice weekly for 12 weeks with MG patients. While the study did not report increased muscle strength but the patient performed significantly better on the physical fitness tests such as the 6-min walk test and 30-second-sit-to-stand test. Another study incorporating respiratory training on stable MG patients for a period of 8 weeks used a case-control approach by randomly dividing patients into cohorts. The patients were trained for diaphragmatic breathing and pursed lips breathing. Compared to the controls and to the individual baseline values, patients had improved respiratory muscle endurance, maximum inspiratory and expiratory pressures, and thoracic mobility [21].

In the international consensus guidance for management of myasthenia gravis: 2020 update [11], for the first time, “the role of physical training/exercise in MG” was identified as the topic of discussion, which shows that more and more experts have begun to pay attention to the exercise rehabilitation and chronic management of MG patients. However, the low quality of evidence with respect to physical training/exercise and its significance in the management of chronic MG led to its noninclusion as an informing recommendation. So, what are the factors that limit the design and completion of high-quality clinical trials on physical exercise in MG? We summarized the following reasons: (1) it is difficult to recruit subjects. The first step in MG clinical trial is to select subjects based on strict and well-defined criteria for inclusion and exclusion, such as age, course of disease, subtype, severity, concomitant diseases, and received interventions. In addition, the low incidence rate of MG further increases the difficulty of recruitment. Each trial will be recruited within a certain time window. Enrolling enough patients within the specified time is a necessary condition for the success of a trial; (2) in order to ensure the real and effective exercise data, trainings need supervision, which limit the activity scope and activity time of subjects and reduce their compliance; (3) prolonged interventions will introduce more influence factors, making prospective study designs difficult; (4) selection criteria for control group is unclear; (5) double-blind trials cannot be designed; (6) absence of clarity or consensus with respect to standard outcomes in MG is missing.

## 3. Application of AI in the Management of Chronic Diseases

### 3.1. Artificial Intelligence Techniques

The basic idea of artificial intelligence (AI) is to simulate human thinking through a computer, that is, the process of perceiving the world, learning constantly, and making decisions. AI is an emerging field in computer sciences which involve human independent application of specifically designed computer algorithms. AI approaches fall into three categories [22]: (1) exploration and discovery of knowledge, this is also known as knowledge discovery in databases (KDD). KDD is primarily used for identifying information validity. It segregates information as relevant/useful and nonrelevant/nonuseful. One of the important steps towards knowledge discovery is data mining. Data mining technology mainly includes decision tree, neural network, regression, association rule, clustering, and Bayesian classifier; (2) learning from knowledge, the method permits learning from the acquired knowledge. Computers can learn on their own without any assistance or intervention from humans. The method is aimed to enable better decision-making and more accurate predictions about future conditions; (3) reasoning from knowledge is the third method. In this method, the existing knowledge becomes the starting point, and using logical techniques, such as deduction and induction, a hypothesis is proved or disproved and helps derive conclusions. For instance, an intelligent medical diagnosis system can diagnose based on the clinical and pathological presentation of the disease, using the knowledge in the databases and the control strategies.

### 3.2. Emerging Application of AI in Medicine

AI was first used in medicine in the 1970s, but the development in the field of medicine has been slow in the following decades. Until the last few years, with the development of information and communication technologies, things have changed. First, the rapid development of software and hardware technology makes it possible to obtain widely available medical data. The popularity of electronic health records (EHR) and the emergence of sensors and wearable devices are promoting the evolution of medical data to digital form. The massive medical data obtained can be processed in real time and efficiently by using big data analysis technology, such as machine learning, deep learning, and data mining. Secondly, the real-time transmission and sharing of information are realized through the Internet. 5G communication technology is about to promote the transformation of mobile Internet to the Internet of everything. The progress and combination of these technologies are driving new developments in the field of medical technology. At present, AI is widely used in health technology. They are utilized at every stage of disease management, including disease screening, [23, 24], disease diagnosis [25–28], prognosis estimation [29, 30], decision support [31], and therapeutic recommendation [32]. Recently, AI attracted considerable interest for its advantages in health and chronic disease management [33, 34].

### 3.3. Examples of AI Applications in Chronic Disease Management

The full name of chronic diseases is chronic noncommunicable diseases (NCDs). The common chronic diseases mainly include cancer, diabetes, hypertension, obesity, chronic respiratory diseases, chronic kidney diseases, autoimmune diseases, cardiovascular and cerebrovascular diseases, and neurodegenerative diseases [35]. Globally, chronic diseases are known to affect nearly a quarter of the adult population and are known to have a huge negative socioeconomic impact [34]. In the following, we will introduce some examples of AI application in chronic disease management and related research.

Chronic disease management requires patient monitoring advice and status assessment. This is one of the application areas to explore AI methods in combination with other mobile computing and sensor technologies, which is expected to create and provide better services for chronic disease management. For instance, in diabetes, AI methods are used for blood sugar monitoring, lifestyle recommendations, and self-management. Exploration of a computerized decision support system (DSS) for diabetes has been undertaken. These computer applications monitor disease outcomes by recording information about diet, exercise, drug use, and blood glucose levels [36, 37]. The utility of such digital technology can also be seen in the management of chronic lung conditions. Res-App is used for monitoring patient breathing through the phone microphone providing an evaluation for several lung diseases, such as asthma, pneumonia, lower respiratory tract disease, croup, and bronchiolitis [38]. Altogether, a combination of sensor-based and computerized technologies either in form of wearable devices or an electronic health record database has helped improve the management of some chronic diseases through the integration of geospatial and clinical data [39, 40].

In cancer research, AI is widely used to evaluate the degree of tumor invasion, predict the course of the disease and prognosis, and give the advice on treatment, especially provides a strong analytical support for the study of breast cancer [41], hepatocellular carcinoma [42], and nasopharyngeal carcinoma [43]. Recently, the US Food and Drug Administration (FDA) has licensed several AI systems to develop testing devices for early diagnosis of cancer [44].

Through the AI algorithm, dozens or even hundreds of groups of data can be analyzed in detail to reveal the inherent laws of diseases and find out the associated factors of the occurrence, development, treatment, and prognosis of some chronic diseases, which is beyond the ability of human beings themselves. A prospective cohort study of 500,000 subjects was completed in the UK. Each subject provided data including biological measurements, lifestyle indicators, biomarkers in blood and urine, and brain imaging information. The researchers also collected genome-wide gene data of all subjects, aiming to look for genetic associations associated with chronic diseases and their characteristics by using big data analysis [45]. Also, in an eight-year study, 109 subjects at high risk of type 2 diabetes received each quarter a measuring and sampling, including the clinical signs measurement, group analysis (genome, immunome, transcriptome, proteome, metabolome, and microbiome), and wearable equipment measurement. Finally, the analysis revealed 67 clinically actionable health discoveries and developed a predictive model for insulin resistance [46]. Without AI-based machine learning algorithms, such huge and complex data analysis was unimaginable in the past. Machine learning is also widely used in the field of nutrition to develop personalized diet management and prevent diet-related diseases [47].

## 4. Application Prospect of AI in Rehabilitation and Management of MG

### 4.1. Establish Monitoring System of Medication Safety and Adverse Events

Long-term treatment with immunosuppressive drugs is essential for most MG patients, and glucocorticoids in particular are irreplaceable. Oral prednisone is recognized as the first-line immunotherapy for MG, but the cumulative exposure dose of prednisone is associated with an increased risk of adverse reactions, including obesity, osteoporosis, abnormal glucose metabolism, and infection. Nonsteroidal immunosuppressants (such as azathioprine, mycophenolate mofetil, cyclosporine, tacrolimus, cyclophosphamide, and methotrexate) are recommended to be used in combination with glucocorticoids or alone for long-term (even lifelong) treatment [10, 11]. Treatment-related adverse event (AE) arising due to immunosuppressive therapy in the initial months should be strictly monitored, such as leukopenia, thrombocytopenia, hepatic dysfunction (particularly with azathioprine treatment), or renal dysfunction (more common with cyclosporine treatment) [48], which has often been neglected in the past. According to statistics, over 90% of AE or serious adverse event (SAE) are not reported in spontaneous reporting systems [49]. Through the digital management platform, an effective utilization of AI technology could send reminders of drug safety monitoring to patients regularly, process the acquired monitoring data in real time, warn patients and doctors of abnormal data, and realize remote monitoring of treatment. Remote management of MG may work to be of much use for the aged patients with travelling difficulties, or in geographically challenged territories, or in times when healthcare facilities are not easily accessible such as pandemic periods.

### 4.2. Conduct Personal Life Guidance and Disease-Related Education

Patients using steroids should be supplemented with calcium and vitamin D and should be given with bisphosphonate therapy appropriately. At the same time, a generally healthy diet should be advised for all patients. It is worth noting that methotrexate and cyclophosphamide should be avoided in women of childbearing age because of their teratogenic effects. Some drugs associated with MG deterioration need to be used with caution and only upon suitable prescriptions from the doctors [48]. The similar notes and related knowledge, including diet, lifestyle, sleep, stress, and exercise habits, should be easily accessible to MG patients at any time on the management platform, and relevant safety warnings should be made according to the data generated by the patients. The management platform should be set up with an education section and constantly updated to improve the patient's self-management skills. It is worth learning from the application of AI in the individualized management of diabetes patients, including AI assisting diabetic patients to make scientific diet plan, carry out appropriate physical exercise, monitor the occurrence and development of common complications, and even provide technical support for patients in blood glucose monitoring and insulin use [21, 32].

### 4.3. Develop a Social Platform for Mutual Benefit between Doctors and Patients

The digital management platform itself also has the social function of communication. The growing partnership and cooperation are the foundation of development. Doctors can recruit the clinical trial subjects, spread knowledge, and demonstrate professionalism through the platform; patients can gain knowledge; understand condition; develop their own social network; read news; and share blogs, photos, and videos.

### 4.4. Participate in the Research Data of MG Rehabilitation and Management

Although, as mentioned above, clinical research on MG rehabilitation is currently facing difficulties, and there seems to be an opportunity to break through this bottleneck with the continuous maturity of AI technology and the development of information and communication technologies. First, AI will be applied to subject recruitment. After the establishment of MG management platform, patient information will be stored and accumulated and, gradually, developed into a valuable clinical resource database. When a study needs to recruit subjects, AI can preliminarily screen and match qualified subjects according to the inclusion and exclusion criteria and, then, recommend candidate subjects to doctors and provide their contact information so as to improve the efficiency of recruitment, expand recruitment coverage, and influence and achieve the best match. Instead of making regular trips to the hospital to participate in clinical trials, patients will receive remote monitoring, treatment, and life guidance. This AI-based clinical trial matching system has been successfully tested at the Mayo Clinic [50]. AI will provide more benefits to the subjects and improve their compliance. For example, AI can provide doctors with automatic, continuous, and real-time monitoring information from subjects through the management platform, so that subjects can get more attention from doctors.

In addition, wearable sensors and video surveillance can be used to automatically and continuously collect patient data, which is becoming increasingly available through the introduction of App-based and sensor-based exercise and therapy management systems [51]. For example, a trial of telerehabilitation after stroke [52] and a study in Parkinson's disease [53], both used data from sensors that measured body movement. For MG, we propose to establish a digital management platform that allows patients in consultation with physiotherapists to allocate patient-specific physical regimen to either the patient or the caregiver. The mobile technology may also allow the collection of adherence data. This real-time collection of data enables to minimized loss of data and ensures volume, variety, and velocity of data collection. The three V's of big data era will allow trend detection and correlations through the application of big data analytic tools. In the future, when enough available data are obtained, big data technologies will use all types of full data (not sample data) to draw reliable statistical conclusions, instead of extrapolating the real world around a small sample of data.

## 5. Conclusion

MG is an incurable disease. Rehabilitation and individualized patient management are essential components of MG lifetime therapy. In the future, drawing on the successful experience of AI in other chronic disease management, it is an unstoppable trend to develop MG digital management platform with functions such as education, management, social contact, and access to research data.

## Data Availability

This article has no additional data.

## Abbreviations

AE: adverse event
AI: artificial intelligence
CSR: complete stable response
EHR: electronic health records
FDA: Food and Drug Administration
IVIg: intravenous immunoglobulin
MG: myasthenia gravis
MGFA: Myasthenia Gravis Foundation of America
MMS: minimum performance state
NCDs: noncommunicable diseases
SAE: serious adverse event.
